# Integrative proteomic analysis of the NMDA NR1 knockdown mouse model reveals effects on central and peripheral pathways associated with schizophrenia and autism spectrum disorders

**DOI:** 10.1186/2040-2392-5-38

**Published:** 2014-07-04

**Authors:** Hendrik Wesseling, Paul C Guest, Chi-Ming Lee, Erik HF Wong, Hassan Rahmoune, Sabine Bahn

**Affiliations:** 1 Department of Chemical Engineering and Biotechnology, University of Cambridge, Tennis Court Road, Cambridge, CB2 1QT, UK; 2 AstraZeneca Pharmaceuticals, 1800 Concord Pike, Wilmington, DE 19850, USA; 3 Department of Neuroscience, Erasmus Medical Center, Rotterdam, CA, 3000, The Netherlands

**Keywords:** ApoA1, Glutamate, Leptin, Major depressive disorder, Oligodendrocytes, Proteomics, Serum biomarkers, SRMstats

## Abstract

**Background:**

Over the last decade, the transgenic N-methyl-D-aspartate receptor (NMDAR) NR1-knockdown mouse (NR1^neo−/−^) has been investigated as a glutamate hypofunction model for schizophrenia. Recent research has now revealed that the model also recapitulates cognitive and negative symptoms in the continuum of other psychiatric diseases, particularly autism spectrum disorders (ASD). As previous studies have mostly focussed on behavioural readouts, a molecular characterisation of this model will help to identify novel biomarkers or potential drug targets.

**Methods:**

Here, we have used multiplex immunoassay analyses to investigate peripheral analyte alterations in serum of NR1^neo−/−^ mice, as well as a combination of shotgun label-free liquid chromatography mass spectrometry, bioinformatic pathway analyses, and a shotgun-based 40-plex selected reaction monitoring (SRM) assay to investigate altered molecular pathways in the frontal cortex and hippocampus. All findings were cross compared to identify translatable findings between the brain and periphery.

**Results:**

Multiplex immunoassay profiling led to identification of 29 analytes that were significantly altered in sera of NR1^neo−/−^ mice. The highest magnitude changes were found for neurotrophic factors (VEGFA, EGF, IGF-1), apolipoprotein A1, and fibrinogen. We also found decreased levels of several chemokines. Following this, LC-MS^E^ profiling led to identification of 48 significantly changed proteins in the frontal cortex and 41 in the hippocampus. In particular, MARCS, the mitochondrial pyruvate kinase, and CamKII-alpha were affected. Based on the combination of protein set enrichment and bioinformatic pathway analysis, we designed orthogonal SRM-assays which validated the abnormalities of proteins involved in synaptic long-term potentiation, myelination, and the ERK-signalling pathway in both brain regions. In contrast, increased levels of proteins involved in neurotransmitter metabolism and release were found only in the frontal cortex and abnormalities of proteins involved in the purinergic system were found exclusively in the hippocampus.

**Conclusions:**

Taken together, this multi-platform profiling study has identified peripheral changes which are potentially linked to central alterations in synaptic plasticity and neuronal function associated with NMDAR-NR1 hypofunction. Therefore, the reported proteomic changes may be useful as translational biomarkers in human and rodent model drug discovery efforts.

## Background

The transgenic NR1-knockdown (NR1^neo−/−^) mouse constitutively expresses only 5 to 10% of the essential N-methyl-D-aspartate receptor (NMDAR) NR1 subunit [1]. The NMDAR is crucial in neuronal development and physiology, and decreased levels or altered function of this receptor have been associated with the pathophysiology of schizophrenia (SZ) [2-5]. Consequently, the NR1^neo−/−^ mouse has been widely used as a genetic model for intrinsic NMDAR hypofunction in preclinical drug discovery efforts. NR1^neo−/−^ mice display hyperlocomotion and increased stereotypic behaviour, which represent standard behavioural readouts for the evaluation of animal models of SZ. These behavioural effects can be attenuated by the typical antipsychotic drug haloperidol, a potent highly specific D_2_-dopamine receptor antagonist [6], and by the atypical antipsychotic drug clozapine [1], which affects a broader spectrum of neurotransmission systems [7,8]. In addition to these behavioural changes, NR1^neo−/−^ mice also show significant impairments in spatial cognitive performance [9], reduced social interaction, escape behaviours, and actively avoid interaction with intruder males. Furthermore, NR1^neo−/−^ males have been reported to be infertile due to their abnormal social behaviour. However, administration of clozapine has been found to ameliorate all of these symptoms [1], which are thought to predominantly reflect the negative and cognitive symptoms of SZ.

Interestingly, recent studies investigating the NR1^neo−/−^ mouse identified behavioural and electrophysiological deficits relevant to all core symptoms of autism spectrum disorders (ASD) [10,11]. Further, clinical ASD symptomatology, including reduced prepulse-inhibition, auditory-evoked response N1 latency, and reduced gamma synchrony was observed in the NR1^neo−/−^ mouse [12]. NMDAR NR1 subunit knockout in parvalbumin-positive interneurons resulted in an ASD-like phenotype [13] with impaired self-care and sociability [14] in the absence of depression-related behaviours [15]. In addition, NMDAR and glutamate abnormalities have been identified in various brain disorders, such as major depressive disorder [16,17] and ASD [18-20], which are characterized by negative symptom domains. The abovementioned behavioural data now supports the hypothesis of a potential role of impaired NMDAR function in the continuum of negative symptom phenotypes of a range of psychiatric disorders, including core features of autism [21].

Given these similarities, the primary objective of this study was to identify molecular signatures in the NR1^neo−/−^ mouse model and to gain insights into the downstream molecular effects of glutamate dysfunction. A combination of three proteomic platforms was employed to explore a wide range of protein abundance changes in brain tissue and serum samples from the NR1^neo−/−^ mouse model. Specifically, multiplex immunoassay profiling was used to assess serum changes given the high sensitivity of this method for quantification of low abundance circulating proteins such as cytokines, hormones, and growth factors. Label-free liquid chromatography – mass spectroscopy in expression mode (LC-MS^E^) analysis was used as this allows unbiased screening of approximately 1,000 proteins in a single extract and targets proteins, such as membrane receptors, nuclear factors, mitochondrial proteins, and cytoplasmic molecules, all of which have been implicated in psychiatric disorders. Finally, SRM mass spectrometry was used to target specific classes of proteins with greater sensitivity than the LC-MS^E^ approach. A secondary goal was to investigate whether changes in protein levels in serum can be linked to glutamatergic brain dysfunction, thus evaluating the translational utility of serum biomarker changes for psychiatric disorders.

## Methods

### Animals

The NR1^neo−/−^ mice [1,22,23] were obtained from the laboratory of Dr. Beverly Koller (The University of North Carolina at Chapel Hill) and a breeding colony was established at AstraZeneca Pharmaceuticals LP (Wilmington DE 19850, USA). All breeding and testing procedures were conducted in strict compliance with the “Guide for the Care and Use of Laboratory Animals” (Institute of Laboratory Animal Resources, National Research Council, 1996) and approved by the Institutional Animal Care and Use Committee of the University of North Carolina and AstraZeneca R&D Montréal. The breeding and genotyping was performed as previously described [24-27]. It involved three populations of mice: NR1^neo+/−^ heterozygotes maintained on C57BL/6 background (Jackson Laboratory), NR1^neo+/−^ heterozygotes maintained on 129/SvEv background (Taconic Farm), and an intercross between female C57BL/6 NR1^neo+/−^ and male 129/SvEv NR1^neo+/−^ to generate the F1 male NR1^*neo−/−*^ and wildtype (WT) mice that were analysed in this study. Homozygotes carrying the NR1 hypomorphic mutation do not breed effectively. Therefore, the mutant homozygotes had to be generated by cross-breeding heterozygotic mice. The NR1 hypomorphic mutation could not be induced in pure C57BL/6 J or 129SvEv mice, because the mutants did not gain weight at the same rate as WT mice and the frequency of mutants born was less than expected. To overcome these problems, the strategy of breeding F1 hybrids was developed to generate the *NR1*^neo/neo^ mice [27]. Therefore, the progeny from the intercross were genetically identical F1 hybrids with the exception at NR1 locus: 50% NR1^neo+/−^, 25% NR1^neo−/−^, and 25% WT. The following primers were used for genotyping: NR1 (+) fwd primer (intron 20) 5′TGA GGG GAA GCT CTT CCT GT3′; NR1 (−) fwd primer (neo) 5′GCT TCC TCG TGC TTT ACG GTA T3′; and NR1 common reverse primer (intron 20) 5′AAG CGA TTA GAC AAC TAA GGG T3′. Mice were housed on a 12 h light/dark cycle with access to food and water *ad libitum.* Mice (3 to 4 months old) were killed according to schedule, decapitated, and trunk blood was collected in ice-chilled tubes containing EDTA and centrifuged at 1,100 *g*, 4°C, for 15 min. The serum was immediately separated and stored frozen at −80°C for later use. Brains were dissected on ice. Frontal cortex and hippocampus tissue were stored at −80°C.

### Multiplex immunoassay profiling

Serum samples were analyzed using a rodent multianalyte profiling platform comprising multiplexed immunoassays of 75 analytes (Additional file 1: Table S2) in a Clinical Laboratory Improved Amendments (CLIA)-certified laboratory at Myriad-RBM (Austin, TX, USA), as described previously [28]. Immunoassays were calibrated using duplicate standard curves for each analyte and raw intensity measurements converted to protein concentrations using proprietary software. Multiplexed calibrators (eight levels per analyte) and controls (three levels per analyte) are developed to monitor key performance parameters, such as a lower limit of quantification, precision, cross-reactivity, linearity, spike-recovery, dynamic range, matrix interference, freeze-thaw stability, and short-term sample stability are established for every assay as described by the manufacturer (http://www.myriadrbm.com/technology/data-quality/). Data analyses were performed using the statistical software package R (http://www.r-project.org) and the analyte levels were determined. Analyses were conducted under blinded conditions with respect to sample identities and samples were analyzed in random order to avoid any sequential biases.

### Sample preparation

Tissue samples were added to fractionation buffer containing 7 M urea, 2 M thiourea, 4% CHAPS, 2% ASB14, 70 mM DTT, and protease inhibitor at a 5:1 (v/w) ratio [29]. Samples were sonicated (10 sec, 2 cycles) and vortexed at 4°C for 30 min. Samples were then centrifuged at 17,000 *g* at 4°C. Protein concentrations of the lysates were determined using a Bradford assay (Bio-Rad; Hemel Hempstead, UK). Approximately 100 μg was precipitated using acetone. After dissolving the precipitate in 50 mM ammonium bicarbonate, reduction of sulfhydryl groups were performed with 5 mM DTT at 60°C for 30 min and alkylation was carried out using 10 mM iodacetamide, incubated in the dark at 37°C for 30 min, and subsequently digested using trypsin at a 1:50 (w/v) ratio for 17 h at 37°C. Reactions were stopped by the addition of 8.8 M HCl in a 1:60 (w/w) ratio. Quality control samples were prepared to monitor machine and preparation performance.

### Label-free LC-MS^E^ analysis

Brain tissue samples were analysed individually in technical duplicates. Splitless nano-ultra-performance liquid chromatography (UPLC) (10 kpsi nanoAcquity; Waters Corporation, Milford, MA, USA), was coupled online through a New Objective nanoESI emitter (7 cm length, 10-mm tip; New Objective, Woburn, MA, USA) to a Waters Q-TOF Premier mass spectrometer. Data were acquired in expression mode (MS^E^) and the total continuous run time was 8 days. The procedure, quality assessment, and data processing were performed as described previously [30]. LC-MS^E^ data were processed by the ProteinLynx Global Server (PLGS v.2.4 Waters, Milford, MA, USA) for ion detection, extraction, and identification using an ion accounting algorithm [31]. The Swiss-Prot rodent reference proteome database (2011–2013) was used for protein identification searches. To control the false discovery rate (FDR), data were searched against a decoy database, which was the randomised version of the database mentioned above to conserve amino acid frequencies. The FDR was set at the default maximum rate of 4%, as applied before [32-35]. The search parameters were (i) enzyme = trypsin, (ii) fixed modification = carbamidomethylation of cysteines, (iii) variable modifications = oxidation of methionine and phosphorylation at serine, threonine, or tyrosine residues, (iv) initial mass accuracy tolerances = 10 ppm for precursor ions and 20 ppm for product ions, and (v) one missed cleavage allowed. In addition, the following criteria were used for protein identification: (i) ≥3 fragment ions per peptide, (ii) ≥7 fragment ions per protein, and (iii) ≥1 peptide per protein. Raw data and PLGS search results were imported into the Rosetta Elucidator software (build 3.3.0.1.SP3.19, Rosetta Biosoftware; Seattle, WA, USA). Elucidator performed retention time (RT) and mz/charge alignment, feature identification, and extraction for all samples using the Rosetta PeakTeller algorithm. Dynamic background subtraction, smoothing in RT, and m/z dimension and isotopic region creation for peak-matching across all runs were calculated using an RT correction of 4 min at the maximum. A single data file was randomly chosen as the master, and all other sample files were aligned to the master in form of a dynamic RT shift. This procedure allowed the improved identification of peptides and proteins in each sample by taking the available data of all samples into account. Features were filtered for high score and normalized based on total ion current. Only peptides detected in both replicates and in >80% of samples were included in further analysis.

Protein abundance changes were determined using the MSstats package [36] based on linear mixed-effects models on the peptide intensities, following log_2_ transformation and exclusion of intensity values deviating more than three standard deviations from the mean of each group (<1% of total data). Proteins were identified by at least two peptides. The *P* values were adjusted to control the FDR at a cut-off of 0.05 following the Benjamini-Hochberg procedure [37].

### Protein set enrichment analysis

Protein set enrichment analysis was carried out as previously described [38,39]. Significantly changed proteins were partitioned into three bins, according to their predicted fold-change (FC): FC <1.0; FC >1.0, and 1 < FC <1. The R package database org.Mm.eg.db version 2.8.0 was used for gene ontology (GO) term annotation based on entrez gene identifiers. Significant overrepresentation of an annotated GO term per bin was determined by the GOstats package [40]. For each bin, *P* values for the GO categories [41] “biological pathway” and “cellular compartment” were calculated by a conditional hypergeometric test using the entire detected proteome as a background. These tests accounted for the hierarchical structure of the GO terms by first testing the “child terms” of any given GO category and filtering significantly enriched proteins prior to analysis of the “parent terms”, as described previously [42]. This prevented the identification of directly-related GO terms with a considerable overlap of assigned proteins. GO terms with no significant enrichment in any bin (*P* >0.05) and GO terms with less than two annotated proteins were removed. The remaining *P* values greater than 0.05 were replaced by a conservative *P* value of 1. *P* values were replaced by their negative logarithm to the base of ten and then converted to z-scores within their proteomic comparison for every remaining GO term. Finally, one-way hierarchical clustering using “Euclidean distance” as distance function and the “Average Linkage Clustering” method available in the software Genesis [43], was performed on all significantly enriched GO terms. The same enrichment analysis was repeated using KEGG pathway annotation in order to provide an independent *in silico* validation of our findings.

### Label-based selected reaction monitoring (SRM) mass spectrometry

Abundance alterations of a panel of 39 candidate proteins implicated in the pathway analysis of the NR1^neo−/−^ mouse (see results section) were measured using targeted SRM mass spectrometry on a Xevo TQ-S mass spectrometer (Waters Corporation) coupled online through a New Objective nanoESI emitter (7 cm length, 10-mm tip; New Objective) to a nanoAcquity UPLC system (Waters Corporation). The system was comprised of a C18 trapping column (180 μm × 20 mm, 5 μm particle size) and a C18 BEH nano-column (75 μm × 200 mm, 1.7 mm particle size). The separation buffers were (A) 0.1% formic acid and (B) 0.1% formic acid in acetonitrile. For separation of peptides, the following 48-min gradient was applied: 97/3% (A/B) to 60/40% B in 30 min; 60/40% to 15/85% in 2 min; 5 min at 15/85%; returning to the initial condition in 1 min. The flow rate was 0.3 μL/min and the column temperature was 35°C.

SRM assays were developed following a general high-throughput strategy [44]. For method refinement, initially up to 12 unique peptides ranging from 6 to 20 amino acids in length, containing tryptic ends and no miscleavages were chosen for each of the selected proteins. All peptides containing amino acids prone to undergo modifications (e.g., Met, Trp, Asn, and Gln), potential ragged ends, lysine/arginine followed by proline or bearing the NXT/NXS glycosylation motif were generally avoided and only selected when no other options were available [45]. Peptides were checked by Protein BLAST (http://blast.ncbi.nlm.nih.gov/Blast.cgi) searches to ensure uniqueness. For method refinement, up to 12 transitions per peptide were tested in SRM mode. Transitions were calculated using Skyline version 1.2.0.3425 [46] and corresponded to singly charged y-ions from doubly or triply charged precursors, in the range of 350 to 1,250 Da. Transitions were selected based on software internal predictions, discovery proteomics data, and spectral data available through the Human National Institute of Standards and Technology spectral libraries [47]. Method refinement was performed on quality control samples. For the final SRM assays, 2 to 3 peptides with the maximal intensities and highest spectral library similarity (dotp) per protein were selected. A further development step, analysing heavy-label spiked quality control samples in scheduled SRM mode, was used to confirm identity via co-elution, extract the optimal fragment ions for SRM analysis, obtain accurate peptide retention times, and optimize collision energy and cone voltage for the quantification run applying skyline software (MacCoss Lab Software; Seattle, WA, USA) [46]. Heavy labelled forms of these selected peptides (spiketides L) were chemically synthesized via SPOT synthesis (JPT Peptide Technologies GmBH, Berlin, Germany). The final transitions, collision energy, and retention time windows used for each peptide can be found in the supplementary information (Additional file 2: Table S1).

Quantitative SRM measurements comparing NR1^neo−/−^ and WT mice were performed in scheduled SRM acquisition mode with the optimized parameters defined during the assay refinement. For each target peptide a heavy isotope labelled internal standard (JPT Peptide Technologies GmbH) was spiked in the peptide mixture for accurate quantification and identification. All SRM functions had a 2 min window of the predicted RT and scan times were 20 ms, which ensured a dwell time of over 5 ms per transition. Assays were randomly split into three LC-SRM methods using Skyline software. This was done because of scheduling, assay development progress, and assay availability reasons. For each peptide, at least three transitions were monitored for the heavy and light version. Samples were run randomized and blocked [48] in triplicates and blanks and quality control peptide injections (yeast alcohol dehydrogenase, Additional file 2: Table S1) were performed alternating after every biological replicate. Resulting SRM data was analyzed using skyline and protein significance analysis was performed using SRMstats [49]. In the first step, data pre-processing was performed by transforming all transition intensities into log_2_-values. Then a constant normalization was conducted based on reference transitions for all proteins, which equalized the median peak intensities of reference transitions from all proteins across all MS runs and adjusted the bias to both reference and endogenous signals. Protein level quantification and testing for differential abundance among NR1^neo−/−^ and WT mouse groups were performed using the linear mixed-effects model implemented in SRMstats. The scope of validity of our conclusions was restricted to the specific biological replicates in the experiments. Each protein was tested for abundance differences between NR1^neo−/−^ and WT mouse. The *P* values were adjusted to control the FDR at a cut-off of 0.05 according to Benjamini and Hochberg [37].

## Results

### Serum characterisation – Quantitative serum immunoassay profiling

We evaluated the peripheral adaption to the systemically reduced NMDAR-NR1 expression by analysing 75 analytes (Additional file 1: Table S2) in serum using a multiplex immunoassay platform. After principal component analysis data quality assessment and outlier filtering, the analysis resulted in the identification of 29 significantly altered molecules (*P* <0.05) (Table 1). The most prominent changes included a 17-fold increase in apolipoprotein A1 (ApoA1), a 13-fold increase in fibrinogen, and an 8-fold increase in vesicular endothelial growth factor A (VEGF), as well as a 6-fold decrease in insulin-like growth factor 1 (IGF-1). The protein levels of all identified chemokines (Ccl12, Ccl11, Xcl1, Ccl7, and Ccl22) were significantly decreased.

**Table 1:** **Analysis of protein levels in serum of NR1**^**neo−/− **^**(n = 12) and wildtype mice (n = 12) using multiplexed immunoassay**

**A)**						
**Protein Name**	**UniProt ID**	**Gene name**	**Ratio NR1/Wt**		***P***	P*
C-C motif chemokine 12	Q62401	Ccl12	*▼*	0.52	0.0002	0.0060
Insulin-like growth factor I (IGF-I)	P05017	Igf1	*▼*	**0.16**	0.0002	0.0060
Osteopontin (2AR)	P10923	Spp1	*▼*	0.36	0.0003	0.0060
Interleukin-12 subunit alpha (IL-12A)	P43431	IL12A	▲	1.86	0.0004	0.0060
Vascular endothelial growth factor A (VEGFA) – assay 1	Q00731	Vegfa	▲	**7.41**	0.0004	0.0060
– assay 2			▲	**8.26**	0.0006	0.0061
von Willebrand factor (vWF)	Q8CIZ8	Vwf	▲	2.98	0.0005	0.0061
Fibrinogen, alpha polypeptide (Protein Fga)	Q99K47	Fga	▲	**13.19**	0.0007	0.0061
Apolipoprotein A-I (ApoA1)	Q00623	Apoa1	▲	**16.77**	0.0007	0.0061
C-X-C motif chemokine 5 (Cytokine LIX)	P50228	Cxcl5	*▼*	0.75	0.0023	0.0143
Eotaxin (C-C motif chemokine 11)	P48298	Ccl11	*▼*	0.50	0.0024	0.0143
Lymphotactin (C motif chemokine 1)	P47993	Xcl1	*▼*	0.56	0.0025	0.0143
Clusterin (Apolipoprotein J)	Q06890	Clu	*▼*	0.61	0.0028	0.0152
Macrophage colony-stimulating factor 1 (CSF-1)	P07141	Csf1	▲	1.65	0.0031	0.0156
Immunoglobulin A (IgA)	NA	NA	*▼*	0.75	0.0035	0.0158
Glutathione S-transferase Mu 1	P10649	Gstm1	▲	1.57	0.0036	0.0158
Leptin (Obesity factor)	P41160	Lep	*▼*	0.49	0.0051	0.0206
C-C motif chemokine 7 (MCP-3)	Q03366	Ccl7	*▼*	0.51	0.0052	0.0206
Interleukin-1 beta (IL-1 beta)	P10749	IL1B	*▼*	0.58	0.0103	0.0358
Adrenocorticotropic hormone (ACTH)	P01193	Pomc	*▼*	0.58	0.0105	0.0358
Oncostatin-M (OSM)	P53347	Osm	▲	2.15	0.0114	0.0370
Glucagon	P55095	Gcg	▲	1.42	0.0202	0.0612
CC chemokine DC/B-CK (Chemokine (C-C motif) ligand 22)	Q546S6	Ccl22	▼	0.82	0.0210	0.0612
Interleukin-11 (IL-11)	P47873	Il11	▲	1.52	0.0212	0.0612
Epidermal growth factor (EGF) – assay 1	P07522	Egf	▲	2.42	0.0235	0.0654
– assay 2			▲	2.12	0.0287	0.0718
Myeloperoxidase (MPO)	P11247	Mpo	▲	1.53	0.0246	0.0659
Tumor necrosis factor (TNF-alpha)	P06804	Tnf	▲	1.66	0.0283	0.0718
Coagulation factor VII	P70375	F7	▼	0.86	0.0309	0.0748
Neutrophil gelatinase-associated lipocalin (NGAL)	P11672	Lcn2	▲	3.27	0.0443	0.1038
Endothelin-1 (ET-1)	P22387	Edn1	▼	0.72	0.0473	0.1075

The table includes Uniprot ID, gene names, ratios (calculated based on average), *P* values (Mann-Whitney U-test), and adjusted *P* values (P*, Benjamini-Hochberg corrected). Significant analytes with an increase/decrease greater than 5-fold are highlighted in bold.

▼ downregulated, ▲ upregulated.

### Brain characterisation – quantitative LC-MS^E^ proteomic profiling of frontal cortex and hippocampus

**Figure 1:**
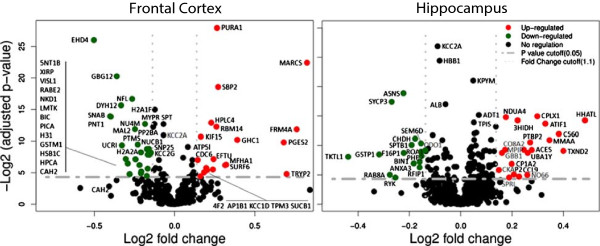
**Volcano plots of group comparisons (10 NR1**^**neo−/− **^**vs. 10 wildtype mice) showing the adjusted significance *P *value (log**_**2**_**) versus fold change (log**_**2**_**).** The plots indicate the most robust protein changes in the NR1^neo−/−^. Horizontal grey lines indicate an adjusted *P* value threshold of 0.05, vertical grey dotted lines indicate a fold-change threshold of 10%. Uniprot-identifiers indicate altered proteins. Significant proteins are quantified by at least two peptides. Full information can be found in Additional file 3: Table S3.

LC-MS^E^ analysis resulted in the identification of 11,345 distinct peptides (563 proteins) in the frontal cortex and 14,775 distinct peptides (883 proteins) in the hippocampus after filtering the data using the criteria described in the Materials and Methods section. In the frontal cortex, 48 proteins were found to be significantly altered by more than 10% (Figure 1, Additional file 3: Table S3). In the hippocampus, 41 proteins showed significant changes using the same criteria.

The EH domain-containing protein 4 (EHD4), adenylosuccinate synthetase isozyme (PURA1), guanine nucleotide-binding protein G(I)/G(S)/G(O) subunit gamma 12 (GBG12), myristoylated alanine-rich C-kinase substrate (MARCS), and selenium-binding protein 2 (SBP2) were identified as most significantly altered in the frontal cortex and synaptonemal complex protein 3 (SYCP3), asparagine synthetase [glutamine-hydrolyzing] (ASNS), NADH dehydrogenase 1 alpha subcomplex subunit 4 (NDUA4), and complexin 1 (CPLX1) were most significantly changed in the hippocampus. CaM kinase II subunit alpha (KCC2A) was detected as highly significantly reduced in both brain regions but showed a 10% decrease.

#### Quantitative LC-MS^E^ proteomic profiling-based pathway analysis

**Figure 2:**
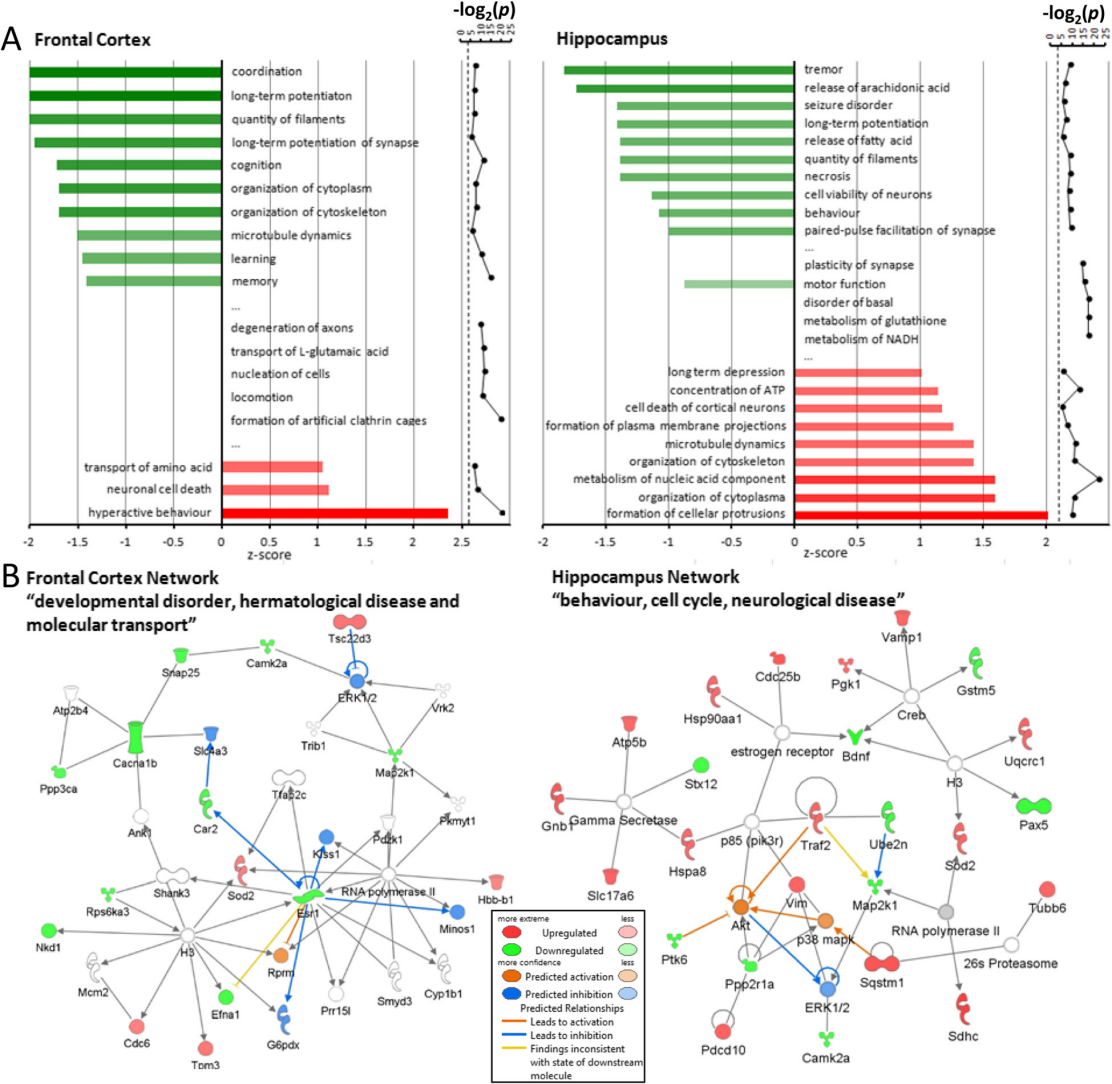
**Computational pathway analysis of brain proteomic profiling. (A)** IPA showing decreased and increased biological functions in NR1^neo−/−^ mouse brain regions. Depicted are functions with an activation score (z-score) >1 (increased activation) or < −1 (decreased activation) with their corresponding log_2_ (*P*) (right graph, the dotted line represents the 0.05 *P* value threshold) as well as the five most-significantly dysregulated non-directionally (z-score > −1, <1, or not predicted) affected pathways (in the middle of the bar plots). **(B)** Identified IPA networks in the data set by global pathway analysis using the Ingenuity Pathways Knowledge Database (IPKB) software. All significantly altered proteins in the frontal cortex and hippocampus from the model were used for the network analysis based on criteria annotated in the IPKB database, which contains molecular information available in the scientific literature. Networks were generated algorithmically on the basis of the connectivity derived from molecular interaction information, scored according to the significant number of focus proteins and assigned associated biological functions (see network descriptions) by overlaying the network molecules onto predefined maps of functional or pathway information in the IPKB database. Proteins are indicated by their gene names. Red and green symbols/text indicate increased and decreased proteins, respectively. Blue symbols indicate predicted inhibition, orange lines indicate predicted activation. Yellow lines indicate inconsistencies with the states of the downstream molecules. Lines ending with arrows indicate activation, lines without arrows indicate interaction/binding.

Ingenuity pathway analysis (IPA) was performed using all significantly changed proteins (P* <0.05) in the frontal cortex (142 proteins) and hippocampus (227 proteins), regardless of the magnitude of change. This assumed that even slight variations in the levels of multiple proteins can result in pathway alterations. Using IPA, the protein changes were assigned to groups of biological functions in the Ingenuity knowledge base and z-scores were calculated as a prediction of whether a biological function was either up- or down-regulated. The biological functions underlying the identified molecular changes in the NR1^neo−/−^ mouse are shown in Figure 2A. The frontal cortex showed a decrease in “coordination”, “long-term potentiation”, and “quantity of filaments”. To a lesser extent, the behavioural domains of cognition, learning, and memory were decreased and hyperactive behaviour was increased. In the hippocampus a broader range of functions appeared to be affected. The most prominent finding here was an upregulation in “formation of cellular protrusions”. Full information including proteins underlying these functions can be found in (Additional file 4: Table S4). Furthermore, we generated functional networks using IPA. Both networks suggested an involvement of the ERK pathway in the two regions. Functional annotation using the ingenuity upstream analysis tool revealed an inhibition of this pathway (Figure 2B).

**Figure 3:**
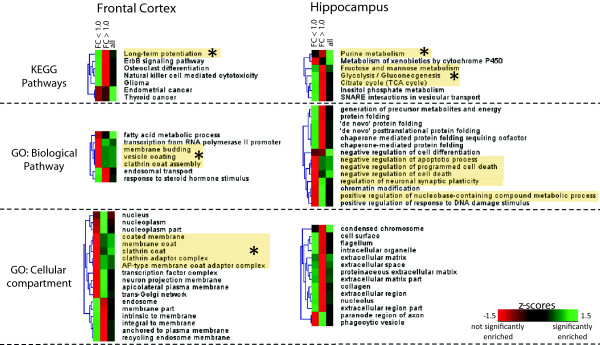
**Functional enrichment analysis of significantly changed proteins in frontal cortex and hippocampus of the NR1**^**neo−/− **^**mouse.** Proteins were divided into fold-change bins for separate analyses. Colour coded z-score transformed *P* values indicate the significance of the enrichment for each bin as indicated. Representative enriched GO terms are annotated. Pathways highlighted in yellow overlapped with the IPA biofunction analysis, asterisks indicate pathways included in the SRM assay validation.

In an attempt to further validate the IPA *in silico* findings, we carried out a GO-term based protein set enrichment analysis. We analysed whether specific GO terms reflecting either biological pathways, KEGG pathways, or cellular compartments were significantly over-represented in the datasets of significantly altered proteins using hypergeometric testing (Figure 3). We validated the involvement of “clathrin adaptor complex/coat assembly/vesicle plasma membrane anchored proteins” and “long-term potentiation” in the frontal cortex, as well as “energy metabolism”, “purine metabolism”, and “apoptosis” in the hippocampus.

#### Validation of significantly changed functional pathways

As a next step, we focussed on the core identified significantly altered pathways and biological functions using SRM, a highly sensitive targeted proteomic method, as an orthogonal validation method of the reported results. We developed an assay panel incorporating proteins involved in the ERK-pathway, clathrin-mediated endocytosis, glutamatergic signal transduction/transport, and energy metabolism. Furthermore, cell-type-specific markers were included. Using SRM we were able to validate most of the significantly altered pathways identified by label-free LC-MS^E^ (Table 2).

**Table 2:** **Significantly changed proteins identified using label-based LC-SRM in the frontal cortex and hippocampus of the NR1**^**neo−/− **^**(n = 12) compared to wildtype mice (n = 12)**

					**Frontal Cortex**						**Hippocampus**					
**Biological Pathway/Function**		**UP-ID**	**Gene name**	**M**	**TPP**	**Ratio NR1/Wt**		***P***	P*	**LC-MS**^**E**^	**TPP**	**Ratio NR1/Wt**		***P***	P*	**LC-MS**^**E**^
**Purine metabolism**																
	Pathway analysis (LC-MS^E^)					*not annotated*					PSEA: ▲ Purine metabolism					
											IPA: ▲ Concentration of ATP					
	Hypoxanthine-guanine phosphoribosyltransferase	P00493	Hprt1	3	5|4	*not significant*					4|4	1.24	▲	0.0054	0.018	
				1	4|5						6|4	1.22	▲	1.8 × 10^-15^	1.4 × 10^-14^	
**Glycolysis/Gluconeogenesis/Tricarbon acid cycle**																
	Pathway analysis (LC-MS^E^)										IPA: Metabolism of NADH					
											PSEA: TCA cycle					
	Aspartate aminotransferase, mito.	P05202	Got2	3	5|4	1.15	▲	3.5 × 10^-05^	4.4 × 10^-04^		5|7	1.15	▲	<×10^-16^	<×10^-16^	
				1	5|7	1.12	▲	8.9 × 10^-16^	1.1 × 10^-14^		5|5	1.18	▲	1.2 × 10^-07^	7.1 × 10^-07^	
	Pyruvate kinase, mito.	P52480	Pkm	3	4|5	1.17	▲	0.0007	0.0059		4|5	1.26	▲	1.3 × 10^-06^	8.3 × 10^-06^	▲***
				2	5|7	1.13	▲	1.4 × 10^-08^	1.6 × 10^-07^		4|7	1.07	▲	0.0028	0.0158	
	NADH-ubiquinone oxidoreductase 75 kDa subunit, mito.	Q91VD9	Ndufs1	1	4|4	1.18	▲	0.001	0.005		8|4	1.28	▲	<×10^-16^	<×10^-16^	
**Neurotransmitter metabolism/transport**																
	Pathway analysis (LC-MS^E^)				IPA: Transport of amino acids							*not annotated*				
					IPA: Transport of L-glutamic acid											
	Proline dehydrogenase 1, mito.	Q9WU79	Prodh	3	5|4	*not significant*					2|2	*not significant*				
				1	2|2						4|3					
	Catechol O-methyltransferase	O88587	Comt	1	4|2	*not significant*					3|3	*not significant*				
	Glutamate decarboxylase 2	P48320	Gad2	2	3|4	1.25	▲	0.0004	0.0015		3|4	*not significant*				
	Vesicular glutamate transporter 1 (VGluT1)	Q3TXX4	Slc17a7	3	5	1.14	▲	0.0031	0.0192		5	*not significant*				
				1	6	1.20	▲	<×10^-16^	<×10^-16^		4|6	*not significant*				
	4-aminobutyrate aminotransferase, mito	P61922	Abat	2	4|6	1.14	▲	0.0006	0.0024		4|5	*not significant*				
**Clathrin-mediated exo-/endocytosis**																
	Pathway analysis (LC-MS^E^)				PSEA: ▲ Vesicle coating, ▲ Membrane budding							*not annotated*				
					PSEA: ▲ Clathrin coated pit etc.											
					IPA: ▲ Formation of artificial clathrin cages											
	AP-2 complex subunit alpha-1	P17426	Ap2a1	1	5|6	1.13	▲	6.5 × 10^-08^	5.2 × 10^-07^		6|6	*not significant*				▲*
	Synaptojanin	Q8CHC4	Synj	2	3|5|3	1.08	▲	0.017	0.041		5|5|5	*not significant*				
	Synapsin-1	O88935	Syn1	2	6|4|3	1.11	▲	0.005	0.016		6|4|3	*not significant*				
	Synaptotagmin-1	P46096	Syt1	2	6|7	1.09	▲	0.001	0.004		5|6	1.11	▲	0.0016	0.0112	▲**
**Neurotransmitter receptors**																
	N-methyl-D-aspartate receptor subunit NR1	P35438	Grin1	3	6	0.26	*▼*	<×10^-16^	<×10^-16^		3|2	0.16	*▼*	<E^-16^	<E^-16^	
	Glutamate receptor 1 (GluR-1)	P23818	Gria1	3	4|3	*not significant*					4	*not significant*				
	Glutamate receptor 2 (GluR-2)	P23819	Gria2	3	5|4|4|4	*not significant*					4|3	*not significant*				
	Glutamate receptor 3 (GluR-3)	Q9Z2W9	Gria3	3	4|2	*not significant*					3|4|3|4	*not significant*				
**Long-term potentiation/Signal transduction**																
	Pathway analysis (LC-MS^E^)				IPA: *▼*Long term potentiation (of synapse)						IPA: *▼* Long term potentiation					
					PSEA: ▼ Long term potentiation											
					PSEA: *▼* EBB-signalling pathway											
	CaM kinase II subunit alpha	P11798	Camk2a	3	3|4	0.90	*▼*	0.0006	0.0056	▼***	3|5	*not significant*				▼*
	CaM kinase II subunit beta	P28652	Camk2b	1	6|6	*not significant*					6|6	0.89	*▼*	1.5 × 10^-08^	1.2 × 10^-07^	
				2	6|6						6|6	0.96	*▼*	3.6 × 10^-05^	1.7 × 10^-04^	
				3	5|4						6|7	0.89	*▼*	1.5 × 10^-08^	1.2 × 10^-07^	
	CaM kinase II subunit gamma	Q923T9	Camk2g	3	4|4	*not significant*				▼***	4|4	*not significant*				
	Calcineurin subunit B type 1	Q63810	Ppp3r1	2	6|6	0.90	*▼*	0.0002	0.001		7|6	*not significant*				
	Ser/thr-protein phosphatase 2B cat. subunit β	P48453	Ppp3cb	2	5|2	1.11	▲	0.012	0.032		5|2	*not significant*				
	Neurochondrin	Q9Z0E0	Ncdn	3	4|3	1.21	▲	0.017	0.083		4|4	*not significant*				
				1	4|3	1.16	▲	0.001	0.005		3|3					▼**
	Disks large homolog 4 (PSD-95)	Q62108	Dlg4	3	5|4	*not significant*					4|5	1.18	▲	0.0004	0.0015	
				1	4|3	1.15	▲	0.0032	0.0099		4|3	*not significant*				
**ERK-Pathway**																
	Pathway analysis				IPA: Implicated in top network						IPA: Implicated in top network					
	Astrocytic phosphoprotein PEA-15	Q62048	Pea15	3	5|4	1.09	▲	0.10	0.33		6|4	1.12	▲	0.007	0.021	▲(*)
				1	8	1.12	▲	0.001	0.005		8|9	1.20	▲	<×10^-16^	<×10^-16^	
	Mitogen-activated protein kinase 1 (ERK-2 )	P63085	Erk2	3	5|5	1.07	▲	0.04	0.13		6|5	1.12	▲	0.0002	0.0009	
				2	4|6|6	1.16	▲	9.8 × 10^-10^	1.7 × 10^-08^		4|6|6	0.94	▲	0.0202	0.0685	
	Mitogen-activated protein kinase 3 (ERK-1)	Q63844	Erk1	3	4|4|4	1.10	▲	0.03	0.11		4|4|4	1.14	▲	0.0004	0.0015	
**Actin cytoskeleton/cell morphology/structural elements**																
	Pathway analysis (LC-MS^E^)	amongst others:			IPA: *▼* Organisation of cytoskeleton						IPA: ▲ Formation plasma membrane projections					
					IPA: *▼* Organisation of cytoplasm						IPA: ▲ Microtubuli dynamics					
					IPA: Degeneration of axons						IPA: ▲ Organisation of cytoskeleton					
					PSEA: ▲ Neuronal projection membrane						IPA: ▲Organisation of cellular protrusions					
											PSEA: ▲ Regulation of synaptic plasticity					
											PSEA: ▲ Neg. regulation of cell differentiation					
											PSEA: ▲ Neg. regulation of cell death/apoptosis					
											PSEA: *▼* Extracellular matrix/space					
	Myristoylated alanine-rich C-kinase substrate	P26645	Marcks	2	6|2|4	1.22	▲	8.4 × 10^-05^	5.7 × 10^-04^	▲***	4|4|6	1.41	▲	1.1 × 10^-08^	1.1 × 10^-07^	▲(*)
				3	4|4|6	*not significant*					6|3|4	1.32	▲	2.3 × 10^-10^	8.0 × 10^-09^	
	PKC and casein kinase substrate in neurons protein 1	Q61644	Pacsin1	3	6|4	*not significant*					6|3	1.28	▲	2.9 × 10^-09^	3.6 × 10^-08^	▼**
	Methionine aminopeptidase 2 (MAP 2)	O08663	Metap2	3	5|4	*not significant*					5|3	1.28	▲	1.6 × 10^-07^	1.1 × 10^-06^	
	Neurofilament light polypeptide (NF-L)	P08551	Nefl	2	3|4|3	*not significant*					3|4|3	1.13	▲	0.0010	0.0086	
	Vesicle-fusing ATPase	P46460	Nsf	3	5|6|6	1.07	▲	0.008	0.047		5|6|5	1.10	▲	0.0005	0.0018	
				2	6|5|4	1.10	▲	2.0 × 10^-07^	1.7 × 10^-06^		6|5|4	*not significant*				
	Neuromodulin (Axonal membrane protein GAP-43)	P06837	Gap43	2	5|6	1.21	▲	0.008	0.022		6	1.21	▲	0.008	0.038	
	Neural cell adhesion molecule 1 (N-CAM-1)	P13595	Ncam1	2	6|5|3	1.11	▲	0.0003	0.001		6|5|3	1.11	▲	0.0003	0.003	
	2’,3’-cyclic-nucleotide 3’-phosphodiesterase (CNPase)	P16330	Cnp	3	5|3|3	0.84	*▼*	0.024	0.108		4|3|5	*not significant*				▼*
	Myelin basic protein (MBP)	P04370	Mbp	3	5	0.87	*▼*	1.7 × 10^-05^	0.0003		5	*not significant*				
				2	5	*not significant*					5	0.93	*▼*	0.0127	0.0479	
	Myelin proteolipid protein (PLP)	P60202	Plp1	3	5|4|4	0.87	*▼*	1.2 × 10^-07^	3.0 × 10^-06^	▼***	5|4|4	1.11	▲	0.0001	0.0005	▼*
				2	6|7|7	0.87	*▼*	<×10^-16^	<×10^-16^		6|7|6	0.96	*▼*	0.0123	0.0479	
	Glial fibrillary acidic protein (GFAP)	P03995	Gfap	3	3|4	1.23	▲	0.002	0.017		3|4	1.29	▲	1.8 × 10^-06^	9.9 × 10^-06^	▲*
	Coronin-1A (Coronin-like protein A)	O89053	Coro1a	1	5|5	1.11	▲	0.003	0.010		5|5	*not significant*				

IPA, Ingenuity pathway analysis; PSEA, Protein set enrichment analysis; UP-ID, uniprot-ID; M, method (assays were split into three methods); TPP, transitions per peptide; G, specific for glutamatergic neurons; B, specific for GABA-ergic neurons; N, specific for neurons; O, oligodendrocyte specific; A, astrocyte specific; M, microglia specific; ▲, increased, *▼*, decreased. *, **, and *** ≤0.05, 0.01, and 0.001, respectively. *P* values were determined using SRMstats and corrected (P*) to control for multiple hypothesis testing [37].

In the LC-MS^E^ phase of the study, we were unable to detect any of the NMDA receptor subunits for quantitative analysis, most likely due to the lower sensitivity of this approach. However, we were able to detect the NR1 subunit using SRM and this revealed a significant decrease in this protein in both the frontal cortex (ratio = 0.26) and hippocampus (ratio = 0.16). In contrast, no changes in other glutamate receptors (GluR-1, GluR-2, GluR-3) were detected in either tissue. In the frontal cortex, abnormalities in neurotransmitter metabolism and transport was indicated through significant abundance changes in the key enzymes glutamate decarboxylase 2, GABA aminotransferase, and the vesicular glutamate transporter 1. Furthermore, clathrin-mediated endocytosis was found to be increased, oligodendrocytic markers decreased, and long-term potentiation altered. In the hippocampus, an alteration in purine metabolism could be confirmed as well as changes in long-term potentiation. The ERK-pathway appeared to be affected in both frontal cortex and the hippocampus, as suggested earlier through pathway analysis.

## Discussion

Herein, we present the first comprehensive proteomic study characterising central and peripheral changes in the NR1^neo−/−^ mouse model. We employed orthogonal quantitative proteomic approaches to investigate protein alterations in serum that can serve as surrogate or translational biomarkers for decreased NMDAR function. The findings associated with NMDAR hypofunction in hippocampus and frontal cortex brain tissue may aid in the discovery of novel drug targets and in elucidating affected downstream pathways. Currently, animal models are almost exclusively assessed using behavioural readouts, leaving questions as to the underlying cellular and molecular network alterations unanswered.

Using multiplex immuno-profiling to measure peripheral metabolic, neurotrophic, and immunological factors, we initially linked the NMDA-mediated glutamatergic hypofunction to several serum analyte alterations. Interestingly, eight out of the 29 changing proteins (ApoA1, coagulation factor-VII, EGF, IGF-1, leptin, TNFα, VEGF, vWF) have previously been reported as changed in SZ and five (ApoA1, Eotaxin, EGF, Leptin, TNFα,) in ASD biomarker studies [50-54]. This supports the notion that serum changes reflect aspects of the pathophysiology associated with psychiatric disorders; providing evidence for the translational utility of serum biomarker studies and their potential for personalised medicine approaches.

The strongest alteration was a 17-fold increase in levels of the lipid transport protein ApoA1. Although ApoA1 has not been linked to effects on glutamatergic signalling before, it is one of the most robust serum biomarkers in SZ [55,56], despite or whereas or even though this has been mainly found to be decreased in CSF, brain, and peripheral tissues of patients. The reason for this apparent discrepancy may be due to adaptive responses which are specific to the NR1^neo−/−^ mouse model. ApoA1 plays a role in cholesterol transport and has been shown to prevent learning and memory deficits in an Alzheimer’s disease mouse model by attenuating neuroinflammation [57]. We also found similar strong increases in fibrinogen, implicating alterations in the blood coagulation system, as well as VEGF, which is produced by neuronal and glial cells in the developing nervous system and directly stimulates neuronal functions such as neurogenesis and cell survival in culture and *in vivo*[58]. This might be linked to our findings in frontal cortex tissue of increased levels of synaptic proteins, indicating increased neuro- and synaptogenesis, which was confirmed by LC-MS^E^, SRM, and computational pathway analysis (Table 2). Synapse formation, maintenance, and plasticity are critical for the correct function of the nervous system and its target organs. During development, these processes enable the establishment of appropriate neural circuitry. In parallel with the increased synaptic markers, we found an increase in proteins involved in neurotransmitter metabolism and transport in the frontal cortex. These findings provide further evidence for increased excitability and imbalance in the frontal cortex, resembling neurochemical changes which are characteristic of SZ [59,60], ASD [61-64], and other disorders with negative symptom domains. At the mechanistic level, VEGF has been shown to exert its neurotrophic properties by regulating NMDAR activity via the SRC family kinase (SFK) pathway [65]. The SFK pathway stimulates signalling events in neuronal cell types, including activation of phospholipase C-gamma, AKT, and ERK. We found abnormal ERK signalling in both brain regions. Therefore, further studies are warranted to investigate the connection of NMDAR and VEGF signalling. Our findings suggest that one or more components of the VEGF signalling pathway might constitute a new therapeutic target for the treatment of SZ and potentially other psychiatric disorders.

This is also the first study linking NMDA-mediated glutamate dysfunction to decreased serum levels of IGF-1. At the circulatory level, IGF-I promotes cell differentiation and growth and may also function as an anti-apoptotic agent [66]. Lower levels of IGF-1 have been found in serum of antipsychotic-naive [67] and antipsychotic-treated SZ patients [68], as well as in children with ASD [69-71]. A recent study reported a relationship between negative symptoms and IGF-1 plasma levels in first episode SZ [72]. Centrally, IGF-1 plays a major role in early brain development, neuro- and synaptogenesis, secretion of various neurotransmitters and myelination processes [73-75]. Remarkably, IGF-1 treatment restores synaptic deficits in neurons from 22q11.2 deletion syndrome patients, a syndrome characterized by an increased risk of SZ and ASD [76], as well as in a SHANK3-deficient mouse model of autism [77]. Thus, we suggest that drugs which target the IGF-1 pathway should be evaluated for the treatment of psychiatric disorders associated with impaired glutamate function. One limitation of the multiplex immunoassay profiling stage of the study is the potential bias in the selection and the molecular class assignment of the investigated molecules. These assays were based on commercial availability and therefore only targeted selected classes of regulatory molecules. Therefore, it is possible that a different selection of molecules would lead to different conclusions from those drawn in this study.

Possibly reflecting IGF-1 function, we found an increase in synaptic markers in the frontal cortex and decreased levels of myelin-specific proteins in the frontal cortex and to a lesser extent in the hippocampus (Table 2). Myelin integrity is crucial for functional neuro-circuitry and perturbations in myelin either during or after neuronal development leads to neurological deficits [78]. The findings are consistent with reports of abnormal myelination in BA10 of SZ, BD [79], and ASD patients [71,80]. Interestingly, effects on glutamate signalling have already been linked to oligodendrocyte dysfunction. Rat brains exposed prenatally to the NMDAR antagonist phencyclidine show reduced levels of oligodendrocyte progenitors [81], resulting in fewer differentiated mature oligodendrocytes capable of producing myelin. Our results provide further evidence of the role of glutamate and its receptors in white matter abnormalities and dysfunction in neurodevelopmental and psychiatric disorders.

Serum profiling also identified an overall decrease in chemokines (Table 1) generally associated with an anti-inflammatory status reflective of an anti-oxidative state [82], which is supported by an increase in glutathione S-transferase levels in the NR1^neo−/−^ model. Centrally, chemokine signalling regulates essential processes for the establishment of neural networks such as neuronal migration and axon wiring [83]. Decreased chemokine functioning has been linked to deficits in social interaction and an increased repetitive behaviour phenotype, as reported in ASD and other neuropsychiatric disorders [84]. Furthermore, we detected decreased levels of leptin. Leptin facilitates hippocampal synaptic plasticity via enhanced NMDAR-mediated Ca^2+^-influx [85]. Impairment of this process might contribute to the cognitive deficits by inducing rapid alterations in hippocampal dendritic morphology and synaptic density [85].

The brain proteomic profiling study also highlights a link between NMDAR and purinergic signalling by identifying corresponding alterations in the hippocampus (Table 2 and Figure 3). Purines play a major role in neurotransmission and neuromodulation with their effects being mediated by the purine and pyrimidine receptor subfamilies P1, P2X, and P2Y. Purinergic signalling is associated with learning and memory [86,87] and locomotor activity, in line with the hippocampal specificity observed in our analysis. At a clinical level, it has been shown that antipsychotics, such as haloperidol, chlorpromazine, and fluspirilen, inhibit ATP-evoked responses mediated by P2X receptors [88,89]. Hypotheses of dysfunctional purinergic signalling have been put forward for psychiatric disorders [90] and ASD [91]. Applied to a maternal immune activation mouse model of ASD, anti-purinergic therapy has been found to reverse core social deficits and sensorimotor coordination abnormalities while, at the same time, normalizing ERK1/2 and CAMK2 signal transduction abnormalities [92]. ERK1/2 and CamK2 pathways are essential components of NMDAR-related signal transduction and were found to be increased, resp. decreased in the NR1^neo−/−^ mouse.

The ERK signalling pathway comprises phosphorylation of proteins involved in transcriptional and translational regulation, dendritic arborisation, cellular excitability, long-term potentiation and depression, neuronal survival, synaptogenesis, and neurotransmitter release [93], and our findings indicated that all of these pathways were altered in the NR1^neo−/−^ mouse. Upstream, ERK activation is regulated by the activity of dopamine, serotonin, and glutamate receptors [94], which are modulated by antipsychotics [95]. Antipsychotics have been shown to differentially mediate the ERK cascade *in vitro* and *in vivo*, dependent on cell and tissue type [96-99]. Clozapine differs from all other antipsychotics by recruiting the EGF-receptor to signal to ERK [100,101], which contributes to clozapine’s broad clinical phenotype. Consistent with this, we found evidence for elevated serum levels of EGF. Further evidence for the involvement of ERK signalling in the pathogenesis of psychiatric spectrum is provided by post mortem brain studies [102,103]. The ERK signalling pathway has also been implicated in the mechanism of action of mood stabilizers [104] and social behaviour [105], and extensively for ASD [106,107]. Interestingly, clozapine has also been shown to be efficacious in the treatment of ASD patients [108]. Remarkably, a recent study showed that the transcriptional regulation exerted by a diverse set of ASD-associated genes (FMR1, TSC1, PTEN, etc.) converges on ERK signalling [109].

With this comprehensive proteomic investigation, we found that the knockdown of one single protein can lead to multiple alterations in a range of signalling pathways both in the central nervous system as well as in blood serum. In brain tissue, we found pyruvate kinase to be one of the most robust changes in the NR1^neo−/−^ mouse, consistent with studies showing increased levels of this key glycolytic enzyme in SZ [110] and in a phencylidine rat model [111]. Furthermore, we found decreased levels of CamKIIa, which is associated with cognitive impairment [112]. Another prominent change was a 20 to 30% increase in MARCS in both regions. MARCS, regulated by calcium-calmodulin and PKC signalling, is a filamentous actin-crosslinking protein involved in cytoskeleton remodelling [113]. Hence, it is involved in the maintenance of dendritic spines and contributes to PKC-dependent morphological plasticity [114] and memory function [115,116]. Furthermore, MARCS is specifically degraded in response to intense NMDAR stimulation. Since the NR1^neo−/−^ mouse expresses only 10% of the NR1 subunit, less NMDAR can be stimulated compared to the WT. This is consistent with our results of increased MARCS levels in the NR1^neo−/−^ model [117]. MARCS has been implicated in proteomic studies of SZ patients [111] and decreased MARCS levels have been associated with both lithium and valporate treatment [118].

## Conclusions

In summary, our results provide the first proteomic characterization of the NR1^neo−/−^ mouse model to date, investigating both brain and serum changes associated with NMDAR hypofunction. We provide evidence for a strong link of neurotransmitter dysfunction and changes in circulating bioactive peptides and proteins, which are implicated in altered brain function and synaptic remodelling. The results presented here provide novel insights into the molecular consequences of altered NMDAR function, such as SZ and ASD, and into the assumed disease mechanisms of psychiatric disorders in which perturbations of NMDAR function are likely to play an important role.

Taken together, the current findings provide further support that neuropsychiatric disorders present with prominent systemic changes affecting a wide range of tissues outside the brain which could represent diagnostic and surrogate markers for personalised medicine approaches in the field of psychiatry.

## Abbreviations

ASD: Autism spectrum disorders; FDR: False discovery rate; GO: Gene ontology; NMDA: N-methyl-D-aspartate; NMDAR: NMDA-receptor; LC-MS^E^: Liquid chromatography–mass spectrometry in expression mode; MARCS: Myristoylated alanine-rich C-kinase substrate; NR1^neo−/−^mouse: NMDA-receptor NR1 subunit knockdown mouse; SRM: Selected reaction monitoring; SZ: Schizophrenia; UPLC: Ultra-performance liquid chromatography; WT: Wildtype.

## Competing interests

SB is a consultant for Myriad-RBM. This does not affect policies regarding sharing of data and materials specified by this journal. None of the other authors declare a conflict of interest.

## Authors’ contributions

HW carried out the label-free LC-MS^E^ experiments, designed and carried out the SRM experiments, and performed all statistical and bioinformatic data analyses. HW prepared the figures and tables and drafted the manuscript. SB, CML, and EHFW conceived the study and participated in its design and coordination. SB, PCG, and HR helped to interpret the results and drafted and edited the manuscript. All authors read and approved the final manuscript.

## Supplementary Material

Additional file 1: Table S2 Full information for all analytes measured using multiplexed immunoassay profiling. Click here for file

Additional file 2: Table S1 Full information for all transitions measured in the multiplexed SRM-assay. Click here for file

Additional file 3: Table S3 Biological classification of differentially expressed proteins identified in the frontal cortex and hippocampus of the NR1^neo−/−^ mouse. Click here for file

Additional file 4: Table S4 Information Ingenuity Pathway Analysis (IPA). Click here for file
