# Supplementary material for: Integrative proteomic analysis of the NMDA NR1 knockdown mouse model reveals effects on central and peripheral pathways associated with schizophrenia and autism spectrum disorders
Source: Mol Autism. 2014 Jul 4;5:38. doi: 10.1186/2040-2392-5-38 (PMC4109791; doi:10.1186/2040-2392-5-38)
Supplement: Additional file 3: Table S3 — Biological classification of differentially expressed proteins identified in the frontal cortex and hippocampus of the NR1neo−/− mouse. [file 2040-2392-5-38-S3.doc]

**Table S3:** Biological classification of differentially expressed proteins identified in the frontal cortex and hippocampus of the NR1^neo-/-^ mouse (ratio cut-off, >1.1 - <0.90; *p** <0.05). PC= peptide count (≥2)). *P*-values were determined using MSstats and corrected (*p**) to control for multiple hypothesis testing (Benjamini-Hochberg, 1995). RG = Regulation of gene expression, epigenetic.

| **Frontal Cortex** | | | | | | |  | **Hippocampus** | | | | | | |
| --- | --- | --- | --- | --- | --- | --- | --- | --- | --- | --- | --- | --- | --- | --- |
| **Biological process** | **Protein Description** | **Swiss Prot ID** | **Gene name** | **PC** | **Ratio** | ***p**** |  | **Biological process** | **Protein Description** | **Swiss Prot ID** | **Gene name** | **PC** | **Ratio** | ***p**** |
| **Cell communication (GO:0007154) & Signal transduction (GO:0007165)** | 3'(2'),5'-bisphosphate nucleotidase 1 | BPNT1 | Bpnt1 | 2 | 0.76 | 6.8E-05 |  | **Cell communication (GO:0007154) & Signal transduction (GO:0007165)** | Annexin A3 | ANXA3 | Anxa3 | 2 | 0.88 | 8.1E-03 |
|  | CaMK-II subunit gamma | KCC2G | Camk2g | 19 | 0.90 | 4.0E-03 |  |  | Myc box-dependent-interacting protein 1 | BIN1 | Bin1 | 4 | 0.89 | 6.7E-03 |
|  | Cell division control protein 6 homolog | CDC6 | Cdc6 | 5 | 1.10 | 7.7E-03 |  |  | Guanine nucleotide-binding protein G(I)(S)(T) subunit β-1 | GBB1 | Gnb1 | 15 | 1.12 | 2.3E-03 |
|  | EH domain-containing protein 4 | EHD4 | Ehd4 | 2 | 0.71 | 1.5E-08 |  |  | M-phase inducer phosphatase 2 | MPIP2 | Cdc25b | 6 | 1.13 | 1.6E-03 |
|  | Guanine nucleotide-binding protein G(I)(S)(O) subunit γ-12 | GBG12 | Gng12 | 4 | 0.78 | 8.0E-07 |  |  | Ras-related protein Rab-6A | RAB6A | Rab6a | 3 | 1.17 | 3.9E-02 |
|  | Neuron-specific calcium-binding protein hippocalcin | HPCA | Hpca | 2 | 0.86 | 3.3E-03 |  |  | Ras-related protein Rab-8A | RAB8A | Rab8a | 4 | 0.83 | 3.1E-02 |
|  | Hippocalcin-like protein 4 | HPCL4 | Hpcal4 | 2 | 1.17 | 1.3E-04 |  |  | Tyrosine-protein kinase RYK | RYK | Ryk | 5 | 0.84 | 4.3E-02 |
|  | Kinesin-like protein KIF15 | KIF15 | Kif15 | 2 | 1.12 | 6.0E-04 |  |  | Semaphorin-6D | SEM6D | Sema6d | 4 | 0.90 | 7.7E-04 |
|  | Serine/threonine-protein kinase LMTK2 | LMTK2 | Lmtk2 | 2 | 0.82 | 3.7E-02 |  |  | SPARC-like protein 1 | SPRL1 | Sparcl1 | 6 | 1.14 | 4.2E-02 |
|  | Protein naked cuticle homolog 1 | NKD1 | Nkd1 | 5 | 0.87 | 2.8E-02 |  |  | Synaptonemal complex protein 3 | SYCP3 | Sycp3 | 2 | 0.83 | 5.8E-06 |
|  | Nucleobindin-1 (CALNUC) | NUCB1 | Nucb1 | 3 | 0.89 | 2.5E-03 |  | **Cell growth and/or maintanance (GO:0008151)** | Cytoskeleton-associated protein 2 | CKAP2 | Ckap2 | 2 | 1.11 | 1.8E-02 |
|  | Clathrin coat assembly protein AP180 | AP180 | Snap91 | 7 | 1.12 | 4.6E-02 |  |  | Collagen alpha-2(VIII) chain | CO8A2 | Col8a2 | 2 | 1.19 | 1.6E-03 |
|  | Serine/threonine-protein kinase tousled-like 2 | TLK2 | Tlk2 | 2 | 0.89 | 2.3E-03 |  |  | Keratin, type II cytoskeletal 1b | K2C1B | Krt77 | 3 | 2.39 | <1E-16 |
|  | TRAF3-interacting JNK-activating modulator | T3JAM | Traf3ip3 | 2 | 0.84 | 7.1E-03 |  |  | Spectrin beta chain, erythrocytic | SPTB1 | Sptb | 3 | 0.88 | 9.3E-04 |
|  | Visinin-like protein 1 (VILIP) | VISL1 | Vsnl1 | 10 | 0.90 | 4.3E-03 |  | **Metabolism (GO:0008152) & Energy Pathways (GO:0006091)** | 3-hydroxyisobutyrate dehydrogenase, mito | 3HIDH | Hibadh | 2 | 1.17 | 5.1E-05 |
| **Cell growth and/or maintenance (GO:0008151)(GO:0007016)** | Dynein heavy chain 12, axonemal | DYH12 | Dnah12 | 6 | 0.79 | 1.9E-05 |  |  | Acetylcholinesterase (AChE) | ACES | Ache | 2 | 1.21 | 1.7E-03 |
|  | FERM domain-containing protein 4A | FRM4A | Frmd4a | 2 | 1.69 | 2.7E-04 |  |  | ATPase inhibitor, mitochondrial | ATIF1 | Atpif1 | 2 | 1.26 | 7.6E-05 |
|  | Myristoylated alanine-rich C-kinase substrate | MARCS | Marcks | **2** | 1.77 | 1.8E-07 |  |  | Succinate dehydrogenase cytochrome b560 subunit, mito | C560 | Sdhc | 2 | 1.30 | 2.5E-04 |
|  | Neurofilament light polypeptide (NF-L) | NFL | Nefl | 2 | 0.83 | 9.6E-06 |  |  | Choline dehydrogenase, mitochondrial | CHDH | Chdh | 5 | 0.88 | 4.4E-04 |
|  | Tropomyosin alpha-3 chain | TPM3 | Tpm3 | 2 | 1.18 | 2.2E-02 |  |  | Cytochrome P450 1A2 | CP1A2 | Cyp1a2 | 3 | 1.15 | 8.5E-03 |
|  | Xin actin-binding repeat-containing protein 2 | XIRP2 | Xirp2 | 7 | 0.81 | 6.9E-03 |  |  | Fructose-1,6-bisphosphatase 1 | F16P1 | Fbp1 | 4 | 0.86 | 2.4E-03 |
| **Metabolism (GO:0008152); Energy pathways (GO:0006091)** | Adenylosuccinate synthetase isozyme 1 | PURA1 | Adssl1 | 2 | 1.20 | 3.9E-09 |  |  | Glutathione S-transferase P 1 | GSTP1 | Gstp1 | 2 | 0.80 | 2.8E-03 |
|  | Carbonic anhydrase 2 | CAH2 | Ca2 | 10 | 0.89 | 4.3E-03 |  |  | Methylmalonic aciduria type A homolog, mito. | MMAA | Mmaa | 2 | 1.27 | 5.2E-04 |
|  | Cytochrome c, somatic | CYC | Cycs | 5 | 1.15 | 2.4E-02 |  |  | Methylmalonate-semialdehyde dehydrogenase, mito. | MMSA | Aldh6a1 | 2 | 1.10 | 1.1E-04 |
|  | L-dopachrome tautomerase | TYRP2 | Dct | 2 | 1.62 | 3.6E-02 |  |  | NADH dehydrogenase 1 alpha subcomplex subunit 4 | NDUA4 | Ndufa4 | 2 | 1.13 | 3.5E-05 |
|  | Glutathione S-transferase Mu 1 | GSTM1 | Gstm1 | 5 | 0.86 | 3.3E-02 |  |  | 2-oxoglutarate dehydrogenase, mito. | ODO1 | Ogdh | 7 | 0.91 | 1.9E-03 |
|  | NADH-ubiquinone oxidoreductase chain 4 | NU4M | Mtnd4 | 2 | 0.88 | 1.5E-04 |  |  | 1-phosphatidylinositol 4,5-bisphosphate phosphodiesterase β-3 | PLCB3 | Plcb3 | 2 | 1.65 | 7.1E-12 |
|  | Prostaglandin E synthase 2 | PGES2 | Ptges2 | 2 | 1.60 | 1.1E-03 |  |  | Transketolase-like protein 1 | TKTL1 | Tktl1 | 2 | 0.74 | 3.7E-03 |
|  | Succinyl-CoA ligase [ADP-forming] subunit β, mito | SUCB1 | Sucla2 | 3 | 1.15 | 1.8E-02 |  |  | Thioredoxin domain-containing protein 2 | TXND2 | Txndc2 | 3 | 1.32 | 1.8E-03 |
|  | Cytochrome b-c1 complex subunit Rieske, mito | UCRI | Uqcrfs1 | 3 | 0.80 | 1.6E-03 |  | **Protein metabolism (GO:0019538**) | Asparagine synthetase [glutamine-hydrolyzing] | ASNS | Asns | 3 | 0.86 | 2.1E-06 |
| **Protein metabolism** | Elongation factor Tu, mitochondrial | EFTU | Tufm | 3 | 1.18 | 7.1E-03 |  |  | Rab11 family-interacting protein 1 | RFIP1 | Rab11fip1 | 4 | 0.90 | 1.3E-02 |
| **RG (GO:0040029)** | Phosphatidylinositol-binding clathrin assembly protein | PICAL | Picalm | 7 | 0.89 | 2.0E-02 |  | **Regulation of nucleic acid metabolism (GO:0019219)** | Probable ATP-dependent RNA helicase DDX46 | DDX46 | Ddx46 | 3 | 1.10 | 8.7E-03 |
| **Regulation of nucleobase, nucleoside, nucleotide & nucleic acid metabolism (GO:0019219)** | RNA-binding protein 14 | RBM14 | Rbm14 | 2 | 1.20 | 2.0E-04 |  |  | Prohibitin-2 | PHB2 | Phb2 | 10 | 0.90 | 3.4E-03 |
|  | Parathymosin | PTMS | Ptms | 2 | 0.85 | 1.4E-03 |  |  | Polypyrimidine tract-binding protein 2 | PTBP2 | Ptbp2 | 2 | 1.21 | 6.8E-04 |
|  | Histon H3.1 | H31 | Histh3a | 4 | 0.82 | 1.1E-02 |  |  | Heterogeneous nuclear ribonucleoprotein A1 | ROA1 | Hnrnpa1 | 3 | 0.90 | 2.0E-03 |
|  | Surfeit locus protein 6 | SURF6 | Surf6 | 2 | 1.24 | 1.4E-02 |  | **Transport (GO:0006810)** | Complexin-1 | CPLX1 | Cplx1 | 2 | 1.23 | 3.2E-05 |
|  | Histone H2A type 2-A | H2A2A | Hist2h2aa1 | 4 | 0.89 | 2.3E-03 |  |  |  |  |  |  |  |  |
|  | Histone H2B type 1-C/E/G | H2B1C | Hist1h2bc | 4 | 0.89 | 4.5E-02 |  | **Biological process unknown (GO:0000004)** | C-C motif chemokine 9 | CCL9 | Ccl9 | 2 | 1.15 | 3.0E-02 |
| **Transport (GO:0006810)** | AP-1 complex subunit beta-1 | AP1B1 | Ap1b1 | 8 | 1.15 | 2.4E-02 |  |  | Protein-cysteine N-palmitoyltransferase HHAT-like protein | HHATL | Hhatl | 2 | 1.40 | 5.1E-05 |
|  | Protein MAL2 | MAL2 | Mal2 | 2 | 0.84 | 2.7E-04 |  |  | Protein lunapark | LNP | Lnp | 3 | 0.91 | 1.7E-03 |
|  | Beta-soluble NSF attachment protein | SNAB | Napb | 3 | 0.76 | 6.6E-05 |  |  | Bifunct. lysine-spec. demethylase & histidyl-hydroxylase NO66 | NO66 | No66 | 3 | 1.20 | 3.2E-02 |
|  | Rab GTPase-binding effector protein 2 | RABE2 | Rabep2 | 3 | 0.86 | 1.3E-02 |  |  | Ubiquitin-like modifier-activating enzyme 1 Y | UBA1Y | Ube1ay | 2 | 1.20 | 2.3E-03 |
|  | Mitochondrial glutamate carrier 1 (GC-1) | GHC1 | Slc25a22 | 2 | 1.31 | 8.5E-04 |  |  |  |  |  |  |  |  |
|  | 4F2 cell-surface antigen heavy chain | 4F2 | Slc3a2 | 3 | 1.14 | 3.0E-02 |  |  |  |  |  |  |  |  |
|  | Synaptosomal-associated protein 25 (SNAP-25) | SNP25 | Snap25 | # | 0.90 | 3.5E-03 |  |  |  |  |  |  |  |  |
| **Biological process unknown (GO:00000004)** | Malignant fibrous histiocytoma-ampl. sequence 1 h | MFHA1 | Mfhas1 | 2 | 1.25 | 1.3E-02 |  |  |  |  |  |  |  |  |
|  | Cytosolic 5'-nucleotidase 1B (cN1B) | 5NT1B | Nt5c1b | 2 | 0.85 | 7.2E-03 |  |  |  |  |  |  |  |  |
|  | Selenium-binding protein 2 | SBP2 | Selenbp2 | 2 | 1.21 | 2.6E-06 |  |  |  |  |  |  |  |  |
